# Promoting good policy for leadership and governance of health related rehabilitation: a realist synthesis

**DOI:** 10.1186/s12992-016-0182-8

**Published:** 2016-08-24

**Authors:** Joanne McVeigh, Malcolm MacLachlan, Brynne Gilmore, Chiedza McClean, Arne H. Eide, Hasheem Mannan, Priscille Geiser, Antony Duttine, Gubela Mji, Eilish McAuliffe, Beth Sprunt, Mutamad Amin, Charles Normand

**Affiliations:** 1 Centre for Global Health, Trinity College Dublin, 7-9 Leinster Street South, Dublin 2, Ireland; 2 School of Psychology, Trinity College Dublin, College Green, Dublin 2, Ireland; 3 Centre for Rehabilitation Studies, Medicine and Health Sciences Faculty, Stellenbosch University, P.O. Box 241, Cape Town, 8000, South Africa; 4 SINTEF Technology and Society, P.O. Box 124 Blindern, NO-0314, Oslo, Norway; 5 Department of Health Science, Norwegian University of Science and Technology, Tungasletta 2, 7047 Trondheim, Norway; 6 School of Nursing, Midwifery and Health Systems, Health Sciences Centre, University College Dublin, Belfield Dublin 4, Ireland; 7 Handicap International Fédération, 138, avenue des Frères Lumière, 69008 Lyon, France; 8 Handicap International, 8757 Georgia Avenue, Suite 420, Silver Spring, MD 20910 USA; 9 Nossal Institute for Global Health, Level 4, Alan Gilbert Building, Melbourne School of Population and Global Health, The University of Melbourne, Victoria, 3010 Australia; 10 Ahfad University for Women, P.O. Box 167, Omdurman, Sudan; 11 Health Policy & Management, Trinity College Dublin, Room 0.21, 3-4 Foster Place, College Green, Dublin 2, Ireland

**Keywords:** Health related rehabilitation, Leadership, Governance, Policy, Less resourced settings, Realist synthesis, Delphi study

## Abstract

**Background:**

Good governance may result in strengthened performance of a health system. Coherent policies are essential for good health system governance. The overall aim of this research is to provide the best available scientific evidence on principles of good policy related leadership and governance of health related rehabilitation services in less resourced settings. This research was also conducted to support development of the World Health Organization’s (WHO) Guidelines on health related rehabilitation.

**Methods:**

An innovative study design was used, comprising two methods: a systematic search and realist synthesis of literature, and a Delphi survey of expert stakeholders to refine and triangulate findings from the realist synthesis. In accordance with Pawson and Tilley’s approach to realist synthesis, we identified context mechanism outcome pattern configurations (CMOCs) from the literature. Subsequently, these CMOCs were developed into statements for the Delphi survey, whereby 18 expert stakeholders refined these statements to achieve consensus on recommendations for policy related governance of health related rehabilitation.

**Results:**

Several broad principles emerged throughout formulation of recommendations: participation of persons with disabilities in policy processes to improve programme responsiveness, efficiency, effectiveness, and sustainability, and to strengthen service-user self-determination and satisfaction; collection of disaggregated disability statistics to support political momentum, decision-making of policymakers, evaluation, accountability, and equitable allocation of resources; explicit promotion in policies of access to services for all subgroups of persons with disabilities and service-users to support equitable and accessible services; robust inter-sectoral coordination to cultivate coherent mandates across governmental departments regarding service provision; and ‘institutionalizing’ programmes by aligning them with preexisting Ministerial models of healthcare to support programme sustainability.

**Conclusions:**

Alongside national policymakers, our policy recommendations are relevant for several stakeholders, including service providers and service-users. This research aims to provide broad policy recommendations, rather than a strict formula, in acknowledgement of contextual diversity and complexity. Accordingly, our study proposes general principles regarding optimal policy related governance of health related rehabilitation in less resourced settings, which may be valuable across diverse health systems and contexts.

## Background

Governance of health systems comprises the actions adopted by a society to organize itself to promote the health of its population [1]. Although governance is the least understood component of health systems, it impacts on all other health system functions [2]. Good governance may result in strengthened performance of a health system, including effective delivery of health services, and improved health outcomes [2, 3]. Governance has in recent years transitioned to the fore of the international development agenda, indicating a shift from attention to micro level, project specific objectives to macro level issues of policy-making [4].

Policymakers in less resourced settings are required to know how to most effectively strengthen the performance of health systems [5]. Recent developments in the social model and human rights perspective on disability and rehabilitation require that the complexities of leadership and governance be addressed through a participative, transparent, well-defined and structured framework [2, 6]. In many resource poor settings, however, patchworks of health services and different service providers are prevalent [7], with such fragmentation resulting in increased barriers to accessing health services, provision of poor quality services, inefficient use of resources, duplication of services, and decreased service-user satisfaction [8]. Coherent but flexible policies, which weave together health related human rights and opportunities, are essential to promote good governance and leadership of health systems.

Rehabilitation is central to a health system addressing the needs of its population [9]. Rehabilitation is a valuable resource for persons with disabilities, directly contributing to individual wellbeing as well as the socioeconomic development of the community [10]. Rehabilitation may be defined as ‘a set of measures that assist individuals who experience, or are likely to experience, disability to achieve and maintain optimal functioning in interaction with their environments’ (pp. 96) [11]. Rehabilitation and disability in the broader sense are contested concepts however [12, 13], and therefore their application in healthcare continues to be complex and challenging. Conversely, this dynamic also offers opportunities to create innovative leadership and governance mechanisms.

As stated in the Declaration of Alma Ata [14], rehabilitation services are an essential component of primary healthcare aiming to address the main health issues in the community. Importantly, as advocated by the Community Based Rehabilitation (CBR) Guidelines, the health related aspects of rehabilitation are strongly connected to the broader needs, rights, aspirations and wellbeing of persons with disabilities, including in areas relating to education, livelihood, social and empowerment, to enhance quality of life [15]. As emphasized by the United Nations Convention on the Rights of Persons with Disabilities (UNCRPD) [16], comprehensive rehabilitation services are required in the areas of health, employment, education and social services to support participation and inclusion in the community and all aspects of society.

### Rationale for realist synthesis

The overall aim of this research is to provide the best available scientific evidence on principles of good policy related leadership and governance of health related rehabilitation services in less resourced settings. This research was also conducted to support the development of the WHO Guidelines on health related rehabilitation, positioned in the context of the WHO ‘Framework for Action’ for strengthening health systems [17], which comprises leadership and governance as one of six components.

Our aim is to provide broad recommendations for successful policy related leadership and governance of health related rehabilitation in less resourced settings, rather than to offer a strict formula, which would fail to recognize the diversity and complexity of specific national, regional and local contexts. Healthcare systems may be conceptualized as complex adaptive systems (CAS) [18, 19], which are influenced by many factors, including service delivery, health workforce, information, medical products, vaccines and technologies, financing, and leadership and governance [17]. As emphasized by Best et al. [18], ‘although CAS are complex and unpredictable, they are amenable to guided transformation by applying simple rules that are sufficiently flexible to allow for adaptation’ (pp. 423). Policy recommendations arising from a CAS perspective avoid complicated checklists and specific directions for change; rather, the local context is examined and findings are produced as broad principles of action – in contexts such as X, try Y [18]. Accordingly, through conducting this research, we aim to enable 'guided transformation' of policy for leadership and governance of health related rehabilitation in less resourced settings by proposing 'simple rules' or broad recommendations, which require contextual adaptation due to variation in structures, systems, and resources.

## Methods

**Fig. 1:**
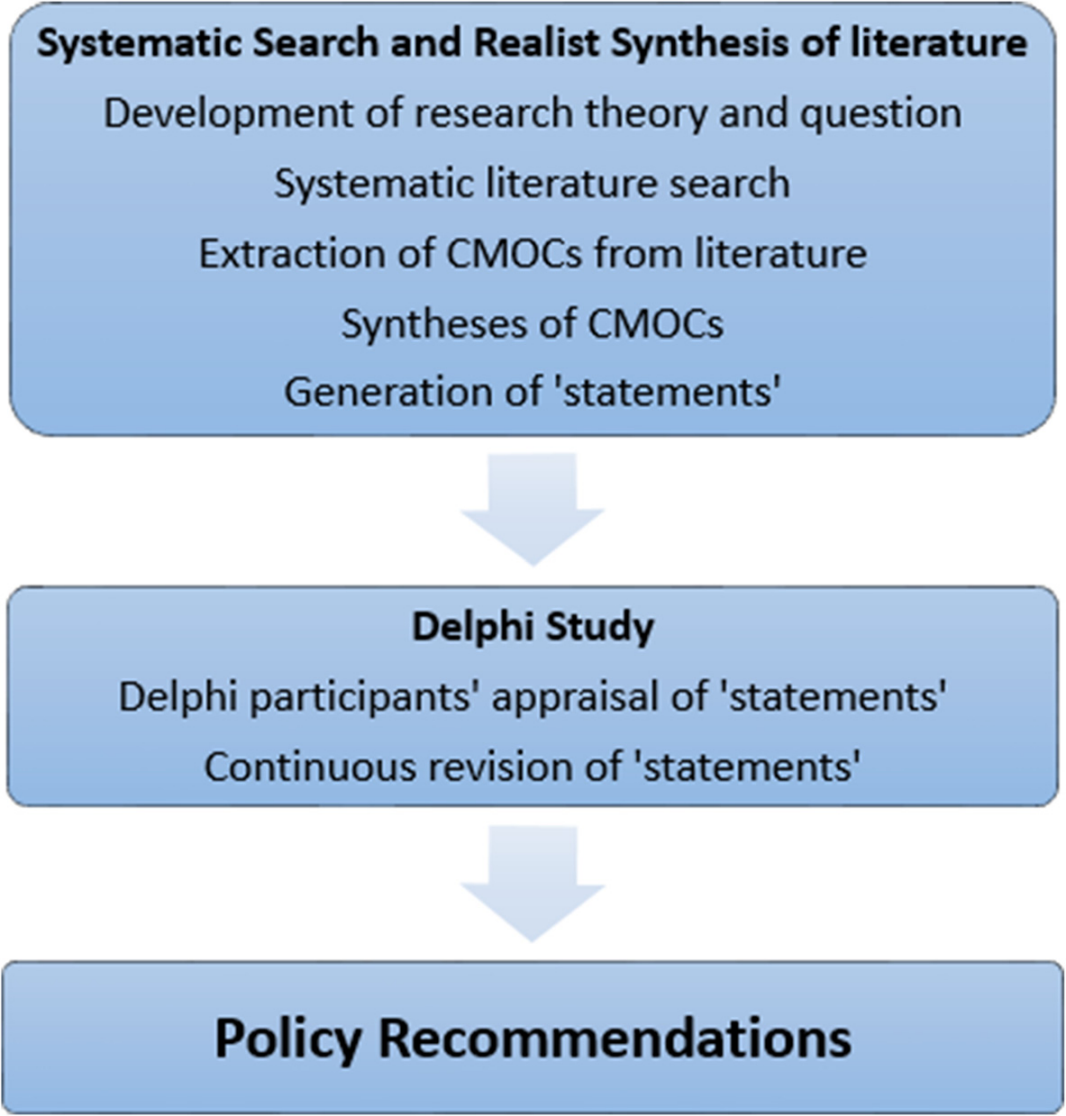
Overview of study methods

This study used two approaches: a systematic search and realist synthesis of the relevant literature, followed by a Delphi survey of the opinions of expert stakeholders on the findings of the realist synthesis. This two stage approach was adopted to combine the authority and contextual focus of a systematic search and realist synthesis of the literature, with the additional value of increased expert stakeholder input provided by the Delphi survey to triangulate, refine and reach consensus on the findings. Outlined in Fig. 1 is an overview of the study methods.

1. Realist synthesis

Governments’ complex assortment of responsibilities and actors indicates that strategies to change national governments’ role in the performance of the health system should not be considered in isolation; rather, these strategies need to be understood in the broader context in which they occur [20]. Accordingly, policymakers are required to understand how and why programmes work and do not work in different contexts, to support their decision-making of which policies or programmes to use and how to adapt them to local contexts [21].

According to Pawson et al. [22], the basic task of the realist synthesis process is to formulate answers to *what* is it about this type of intervention that works, for *whom*, in what *circumstances*, in *whatrespects*, and *why* [23]*.* Realist methods are being increasingly used to explore complex public health issues [24]. Realist syntheses can provide policymakers with rich and pragmatic information with regards to complex health interventions for planning and implementing programmes [22].

Realist approaches assume that nothing works for everyone or in every context; and that context significantly influences programme outcomes, signified by the basic realist formula in Pawson and Tilley’s model of ‘mechanism + context = outcome’ [25]. Pawson et al. [22] argue that understanding what works in social interventions requires establishing causal relationships. In realist inquiry, the cause–effect relationship (for instance as represented by X causes Y) is rigorously explored by trying to determine just how a causal outcome (O) between two events (X and Y) is actually brought about (the mechanism (M)), through the context (C) in which the relationship occurs.

We therefore sought to identify effective patterns and pathways from the contexts, mechanisms, and outcomes of the studies included in the realist synthesis using the context mechanism outcome pattern configurations (CMOCs) formulation of realist synthesis methodology [22].

### Overarching research question

Consistent with realist syntheses [23, 26], a programme theory was created through an iterative process comprising consultation with research team members and exploring relevant literature to formulate a theoretically based evaluative framework for the research question.

As outlined by the WHO [17], ‘leadership and governance involves ensuring strategic policy frameworks exist and are combined with effective oversight, coalition building, the provision of appropriate regulations and incentives, attention to system design, and accountability’ (pp. 3). Good governance from this perspective is policy-centric [5]. Accordingly, the overarching research question was narrowed from ‘leadership and governance’ of health related rehabilitation to focus on policy to provide the most efficient and effective explanatory framework for the research question. A realist synthesis expert confirmed the research method and overarching research question as appropriate and rigorous. The overarching research question is outlined below:

*‘What policies, including processes of policy development, implementation, monitoring and evaluation, promote good leadership and governance of health related rehabilitation in less resourced settings?’*

### Searching process

Both a systematic searching approach and snowballing were used for the literature search, closely following the Cochrane Collaboration Guidelines for conducting a systematic review [27].

A number of sensitive search strategies were initially developed to scope the literature. Based on the number of documents returned from these searches, the search strategy was subsequently refined to a more specific strategy, devised and agreed in collaboration with our research team and with the assistance of a Search Librarian. Using this more refined search strategy, our final search of the literature identified 420 abstracts. Inclusion and exclusion criteria are outlined in Table 1.

**Table 1:** Inclusion and exclusion criteria for realist review

Inclusion criteria	
Publication Year	2003 – present.
Language	No restriction.
	Searching will be conducted in English, with any non-English titles to be translated.
Types of Research	Qualitative, quantitative and mixed methods: - Intervention studies - Descriptive studies
	Research and development studies.
	Programme evaluations.
	Theoretical.
Types of Documents	Primary and secondary (review) studies, including: - Journal articles, book chapters, policy reports, technical reports, conference proceedings and reports, and accessible dissertations. - Commentaries/Editorials
Research Focus	Addresses the following: - Rehabilitation AND leadership/governance with a focus on policy - Low-income setting OR can be applied to a low-income setting
Exclusion criteria	
Publication Year	Prior to 2003.
Types of Research	Protocols.
	Testing measures.
Types of Documents	Book reviews, abstracts, bibliographies.
Research Focus	- Rehabilitation services delivered by different sectors, i.e. vocational rehabilitation - Not applicable to a low-income setting - Non-disability related services
Codes for Exclusion	Rehabilitation – Article does not relate to issues of rehabilitation.
	Policy – Article does not relate to leadership/governance with a focus on policy.
	Setting – Study location not applicable.
	Research – Research method does not fit inclusion criteria.
	Document – Document type does not fit inclusion criteria.

The time filter of 2003 was selected as it was prior to the publication of recent landmark international disability and rehabilitation documents, including the World Report on Disability [11], CBR Guidelines [15], and the UNCRPD [16]. Furthermore, as ascertained during our initial search, this time period reflected a trend of increased relevant publications arising after the year 2003. Search terms are outlined in Table 2.

**Table 2:** Search terms for systematic search of literature

1(a) AND 2 AND 3	
1(b) AND 2 AND 3	
1 (a) Leadership AND policy.	1 (b) Governance AND policy.
2. CAHD OR CBR OR ‘Community approaches to handicap in development’ OR ‘Community based inclusive development’ OR ‘Community rehabilitation’ OR ‘Community based rehabilitation’ OR ‘Functional restoration’ OR Habilitation OR ‘Health related rehabilitation’ OR ILD OR ‘Inclusive local development’ OR ‘Participatory community development’ OR Rehab* OR Rehabilitation OR ‘Restoration of function’ OR (Rehabilitation w/3 (care OR services OR support OR therapy)) OR ((therapy OR therapies) w/3 (cognitive OR complementary OR occupational OR physical OR recreational OR respiratory OR social OR speech)).	
3. Africa OR Asia OR Caribbean OR ‘Central America’ OR ‘Eastern Europe’ OR ‘Latin America’ OR ‘Less resourced’ OR LMIC OR LIC OR ‘Low income countries’ OR ‘Low income country’ OR ‘Low and middle income countries’ OR ‘Low and middle income country’ OR Pacific OR ‘South America’ OR ‘Third world’ OR ((developing OR ‘less developed’ OR ‘least developed’ OR ‘under developed’ OR poor) w/3 (countries or country or nation or nations)).	

Databases were selected as those most relevant to disability, health related rehabilitation, and governance. The following 11 databases were used in the search: PubMed, WHOLIS, Embase, AIM (African Index Medicus), ABI Inform, LILACS, PsycINFO, SCIE, Rehabdata, Scopus, and CIRRIE. A search was also conducted on the archives of the journal ‘Disability, CBR and Inclusive Development’ as these archives were not included in the databases outlined above.

Snowballing comprised emailing organizations outlined by the Office of the United Nations High Commissioner for Refugees [28] and other organizations identified by the research team; contacting team members and other stakeholders to request relevant documents; performing searches on search engines; and searching references of relevant reviews and of all included articles.

### Selection and appraisal of documents

Articles were selected for inclusion in the realist synthesis in numerous stages. At each stage, multiple reviewers from the research team reviewed and selected articles. Articles identified through the databases search were reviewed on article title and, if identified as appropriate, were subsequently reviewed based on abstract and then full text by two researchers from the research team independently. A third reviewer mediated any diverging opinions between the two researchers so that a decision was reached. All appropriate documents from snowballing were reviewed based on full text. Throughout each stage, at least one reviewer had experience in disability and/or rehabilitation and one in health governance/policy.

### Quality rating of articles

The methodological quality of all included articles was assessed using the Mixed Methods Appraisal Tool (MMAT) [29] – a tool designed for the appraisal stage of complex systematic literature reviews that include qualitative, quantitative and mixed methods studies. In accordance with the MMAT, all articles were assigned a score between one and four, whereby one = 25 %, two = 50 %, three = 75 % and four = 100 %, indicating their methodological quality. A score of ‘N/A’ was assigned to articles that could not be appraised as a qualitative, quantitative or mixed methods study.

### Data extraction

For each article, information was collected on CMOCs. A comprehensive, systematic and transparent process of data analysis was developed, involving the design of a data extraction template. Using the template, reviewers extracted CMOCs from each article, and subsequently linked these CMOCs to the research question. Two reviewers, one working in rehabilitation studies and one with a background in disability and policy analysis, independently extracted CMOCs from articles. The reviewers reviewed all included articles and for each article completed as much of the data extraction template as possible according to the information provided by the article. This process was frequently discussed amongst the research team to support the consistency and validity of findings.

### Data syntheses

The primary reviewer synthesized the findings from both reviewers’ CMOCs-extraction of articles. For this process, a data analysis matrix was developed, adapted from a previous realist synthesis [30]. Accordingly, through coding using content and thematic analyses, the primary reviewer identified and synthesized substantial and frequent patterns of CMOCs from both reviewers’ CMOCs.

CMOCs were therefore extracted from the included articles using the data extraction template and synthesized using the data analysis matrix. CMOCs were then grouped into seven themes with a view to contributing to the overarching research question, and developed into statements for the second phase of the research, the Delphi study. The statements were also generated from 30 documents identified through snowballing (for example [16, 31–33]).

2. Delphi study

The methodology for the second phase was a Delphi study. The Delphi study attained ethical approval from the Health Policy and Management/Centre for Global Health Research Ethics Committee of Trinity College Dublin, Ireland. The Delphi survey is a group facilitation technique, which has an iterative, multistage process, designed to convert individual opinion into group consensus [34]. The Delphi aims to achieve consensus on the opinions of experts through a series of structured questionnaires, which are completed anonymously by experts; responses are summarized between rounds and fed back to the participants through a process of controlled feedback, and this process is repeated until consensus is reached [34]. Central to the Delphi method is its anonymity and confidentiality, iterations, controlled feedback, and arithmetic aggregation of group scores [35, 36].

Advantages of the Delphi include providing a mode of group decision-making whereby participants do not need to travel to a group meeting place; anonymity, thereby reducing the impact of social-emotional behavior and allowing participants to focus more so on task oriented activities; and avoidance of direct confrontation between group members [37, 38]. The structure of the Delphi comprises the positive attributes of interacting of interacting groups, including knowledge from diverse sources, while averting their negative components, due to social, personal and political conflicts; it allows input from a substantial number of participants who could conceivably convene in a group meeting, from participants who are geographically dispersed [36].

A panel of experts was recruited, described later in this article, based on experience and expertise in policy and/or rehabilitation, which could provide insight into leadership and governance for health-related rehabilitation. Prior to conducting the Delphi survey, a minimum of 10 participants and a maximum of 25 participants were chosen as the parameters for the sample size of the survey, in accordance with recommendations of sample sizes for Delphi studies [38].

Inclusion criteria for experts were the following: (1) Expert in their field; (2) previous experience working in a less resourced context; (3) previous experience/expertise in the area of leadership and governance; and (4) availability and willingness to participate. Exclusion criteria (criteria additional to not conforming to the inclusion criteria) comprised: (1) Already participating in another Delphi study; and (2) no experience/expertise in areas mentioned in the inclusion criteria.

The panel was recruited through purposeful sampling, specifically snowball sampling. The initial contact list for possible participants was created by the research team. All possible participants identified in the initial list were contacted; if they could not participate, they were asked to suggest other possible participants that fit the criteria. The research team was also included as possible participants in the Delphi as they were considered to be experts. Experts were recruited for the study until sufficient coverage of different categories of experts – service-users, service providers, and policy/decision-makers – was achieved.

For each survey round, participants were emailed with a link to the survey via Survey Monkey [39]. Participants provided their level of agreement and comments in relation to the statements. These comments were used for further adjustments to the statements for the subsequent survey iteration. Statements were rated on a Likert scale ranging from one to five (Strongly Disagree to Strongly Agree). As guided by a previous Delphi Study on health related rehabilitation [40], a statement was considered to be ‘accepted’ or to have reached agreement amongst participants if it attained an average rating of four or above and a standard deviation of below one. Statements that were ‘not accepted’ in a survey round were revised based on participants’ comments, and were put forward to subsequent survey rounds.

## Results

1. Realist synthesis

**Fig. 2:**
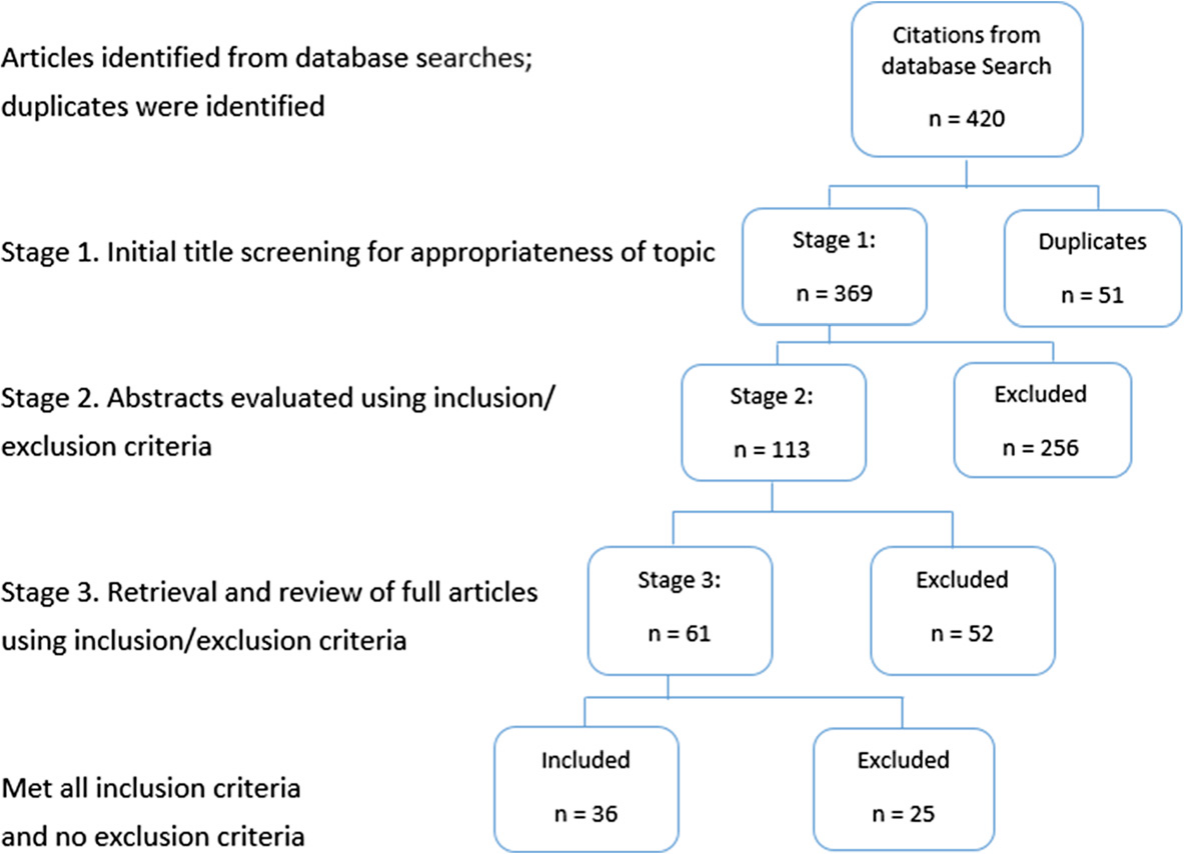
Document flow diagram illustrating the search process

Throughout the databases search, a total of 420 articles were identified. Following the screening process, 36 articles were included in this study, as outlined in Fig. 2. However, six of these documents were larger reports, such as the World Report on Disability [11], and were therefore subsequently excluded with regards to extraction of CMOCs, although these reports provided useful information for explaining and expanding on findings within the context of previous research and theory. An additional six articles were included from a parallel research project [41]. In total therefore, 36 articles were included [42–77].

### Quality of articles

Based on MMAT guidelines, depending on each article’s attributes as a qualitative, quantitative or mixed methods study, one article was scored as one for its methodological quality rating; eight articles were scored as two for their quality ratings; eight articles were scored as three for their quality ratings; and two articles were scored as four for their methodological quality ratings. A further 17 articles were scored as N/A. Therefore, the methodological quality rating of 17 articles could not be assessed, as these articles did not fit the methodological criteria for assessment.

### Context mechanism outcome pattern configurations and statements

**Fig. 3:**
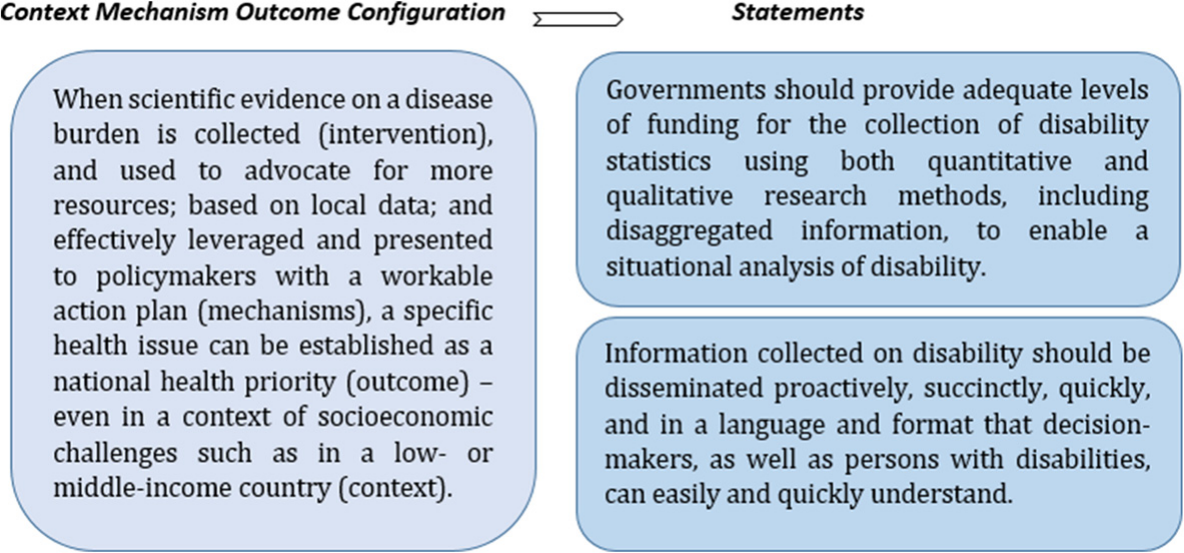
CMOC from a sample included study and its development into statements

To illustrate the process of the extraction and syntheses of CMOCs from the literature, and the development of CMOCs into statements, first, outlined in Table 3 are the synthesized CMOCs for a sample included study. Figure 3 next provides an example of a CMOC synthesized from this study and its development into statements. In total, 51 statements were developed through this process and assessed by participants of the Delphi survey.

**Table 3:** Synthesized CMOCs for a sample included study

Reference: 01 Title: Araya R, Alvarado R, Sepulveda R, Rojas G. Lessons from scaling up a depression treatment program in primary care in Chile. Rev Panam Salud Pública. 2012;32(3):234-240.								
**Key words**	**Setting**	**Design**	**Population**	**Intervention**	**System-wide or project-specific**	**Sectoral or inter-sectoral**	**Cadre**	**Quality (MMAT)**
Community mental health services; Depression; Healthcare delivery; Mental health; Chile.	Chile: Programa Nacional de Diagnóstico y Tratamiento de la Depresión) National Depression Detection and Treatment Program (PNDTD).	Retrospective qualitative study; In-depth semi-structured interviews with six key informants.	Depression treatment programme users.	PNDTD, Chile.	This research reports on a summary of elements that led to scaling up and sustainability of the PNDTD programme, Chile, 2008.	Strategic alliances were created across sectors with strategic partners, between the Mental Health Unit and the Primary Care Division (PCD), and with the Ministry of Women.	Senior Officers at the Ministry of Health (MoH).	3 quality score –Qualitative.
**CMOCs**								
**Contexts**			**Mechanisms**		**Outcomes**	**CMOCs**		
1. Scientific Evidence i) A national disease-burden study was conducted. ii) Two large psychiatric morbidity surveys were conducted. iii) Other studies showed that depression was also very common among primary care patients. iv) A trial was conducted of cost-effectiveness of an improved treatment of depression through primary care in Chile. v) A randomized controlled trial of a programme to improve the management of depressed women in the primary care setting showed positive results. vi) The MoH hired an academic institution to undertake a small scale evaluation of the effectiveness of the programme.			1. i) The psychiatric morbidity surveys were used to advocate for more resources for the PNDTD. ii) The studies were based on local data. iii) The Mental Health Unit at the MoH leveraged available evidence effectively. iv) A workable action plan was presented to policymakers. v) There was ongoing communication between the research team and those designing the programme.		1. The MoH decided that depression would become the country’s third highest health priority for 2002.	1. *Scientific evidence*: When scientific evidence on a disease burden is collected, and used to advocate for more resources; based on local data; and effectively leveraged and presented to policymakers with a workable action plan, a specific health issue can be established as a national health priority – even in a context of socioeconomic challenges such as in a low- or middle-income country.		
2. Teamwork and Leadership i) There was an informal team of leaders acting in parallel at different levels and with a shared vision.			2. Leaders shared common features: “politically friendly” and trustworthy; good at forming alliances; able to apply technical information; and good communicators.		2. Effective teamwork and leadership facilitated the creation of powerful strategic alliances, which facilitated institutionalizing the programme within the ministerial framework.	2. *Teamwork and Leadership*: Effective teamwork and leadership – by a group of respected and “politically friendly” professionals acting as leaders in a team effort; who are capable of communicating effectively with decision-makers; with the capacity to detect emerging opportunities and react accordingly; who are capable of negotiating political agreements at all levels; who have at least basic technical knowledge, and can prepare a solid proposal; and who are trustworthy individuals capable of forming alliances with strategic partners – can create powerful strategic alliances, which can facilitate institutionalizing a programme within a ministerial framework.		
3. Strategic Alliances i) There was a strategic alliance between the Mental Health Unit and the PCD. ii) Other strategic alliances were formed outside of the MoH, with the Ministry of Women and some universities.			3. i) A strong alliance was created – the Mental Health Unit had technical capacity while the PCD had resources. ii) Academics provided information, which provided support for introducing the programme.		3. The PCD accepted ownership and management of the programme.	3. *Strategic alliances*: Strategic alliances – with key individuals who have positions of political power in a MoH; across sectors with strategic partners; that can persist over time; and with other units by which a programme may be co-owned – can result in a PCD accepting ownership and management of a programme.		
4. Programme Institutionalization i) A gradual process occurred of “institutionalization” of the programme.			4. i) The programme was aligned with well-known models of care, similar to those of other ministerial programmes. ii) The programme was introduced as another ministerial programme, complying with regulations and ring-fenced funding. iii) New and ring-fenced funding was secured. iv) A critical-mass of human resources was used. v) The programme had itemized resource allocation, e.g. resource allocation for psychologists, medication, etc. vi) The programme was highly structured in technical and financial terms.		4. The programme was highly sustainable.	4. *Programme institutionalization*: Institutionalizing a programme – by using well recognized models of healthcare delivery within the MoH; placing the programme among other well established PCD programmes; introducing personnel that are widely available and at an affordable cost with the potential to lead the programme locally; and fence-ringing any new and essential financial resources – can result in strong programme sustainability.		
5. Task-shifting: i) Responsibility for most patient care was transferred to the PCD, away from specialized psychiatric services. ii) Transfer of responsibilities from psychiatrists to psychologists was conducted, who were widely available at an affordable price. iii) Psychologists were hired as key players.			5. Task-shifting may increase the availability of human resources, allowing more patients to receive treatment.		5. When the PNDTD was scaled up, psychologists were hired in all primary care centres and became the programme’s cornerstone.	5. *Task shifting:* In contexts of a shortage of specialized health workers, task-shifting to less specialized health workers may increase the availability of human resources for health so that more patients can access healthcare.

2. Delphi study

In total, three rounds of the Delphi survey were conducted. Overall, 19 participants were emailed with the link to the online survey, with 18 participants overall completing all three survey rounds. Twelve participants were female and six were male. Persons with disabilities were represented in the survey with six participants identifying themselves as having a disability. Overall, ten participants were in the 35–44 age group, five participants in the 45–54 group, two participants in the 55–64 group, and one participant in the 75+ age group. Participants’ countries of origin were varied, comprising Egypt (one participant), Nepal (one), India (two), Sri Lanka (one), Pakistan (one), Fiji (one), Australia (one), Britain (three), Ireland (two), Italy (one), France (one), Norway (two), and the Netherlands (one).

A large range of expertise was covered by participants and while each participant was selected for their expertise in one particular area, many had extensive knowledge and experience in more than one relevant area. Years of experience that experts had in their relevant fields ranged from 8 to 55+ years with an average of 18.2 years of experience. Disciplines with which participants identified were as follows: *Human rights*; *disability rights*; *disability and human rights*; *disability law and policy*; *political science and disability*; *health systems*; *health policy*; *health*; *CBR coordinator*; *management*; *epidemiology*; *social development and disability*; *social sciences (disability)*; *governance and social inclusion*; *social sciences*; *physiotherapy*; *medical anthropology*; *community based rehabilitation*; *physical medicine and rehabilitation*; *public health*; *management in non-governmental organization (NGO) in disability and development*; *occupational therapy*; *disability-inclusive development*; *and disability and rehabilitation.* Participants reported experience working in a variety of regions, including Sub-Saharan Africa, North America, and South East Asia.

Participants comprised ***service-users*** including organizations of persons with disabilities (DPOs) (two participants), persons with disabilities (one), and civil society (one); ***service providers*** including physical rehabilitation specialists (one), and a CBR programme manager/coordinator (one); and ***policy/decision-makers*** including NGOs (three), Department of Health (one), policymakers (two), CBR experts (three), and policy analysis experts (three). The participant categories of service-users, service providers, and decision-makers were based on a health related rehabilitation framework published by Handicap International [78].

In the first survey round, 44 statements were considered ‘accepted’ by achieving the criteria for agreement and seven statements were ‘not accepted’; in the second round, 39 statements met the criteria, and 12 statements did not; while in the third and final round, 34 statements met the criteria while 17 statements did not achieve the criteria for agreement.

Using CMOCs developed throughout the realist synthesis, which were subsequently developed into statements and put forward to the Delphi survey, 51 statements emerged as recommendations for policy for leadership and governance of health related rehabilitation in less resourced settings. In total, 34 of these statements were ‘accepted’ by Delphi participants, while 17 statements were scored as ‘not accepted’. Importantly, however, all 51 of the final statements, including the 17 statements that did not meet the criteria for acceptance due to a standard deviation of one or higher, achieved an average score of above four (Agree). The 51 statements, or policy recommendations, are outlined in Table 4, alongside examples of their proposed outcomes. Several broad principles emerged from the research findings:

- *Participation of persons with disabilities in policy processes*, and the research that guides such processes, to improve programme responsiveness, efficiency, effectiveness, and sustainability, and to strengthen service-user self-determination and satisfaction.
- *Collection of disaggregated disability statistics*, and development of health information systems, to enable a situational analysis of disability for the purposes of supporting political momentum, decision-making of policymakers, evaluation, accountability, and equitable allocation of resources.
- *Explicit recognition in policies that disability may interact with other vulnerability factors*, for example displaced populations with disabilities, which may create double discrimination, multiple disadvantages, and increased barriers to accessing health services. Accordingly, *explicit promotion in policies of access to services for all subgroups of persons with disabilities and service-users* to support equitable and accessible services.
- *Strong inter-sectoral coordination for the provision of rehabilitation services*, including CBR, for the purposes of creating coherent mandates across governmental departments regarding service provision.
- * ‘Institutionalizing’ rehabilitation programmes by aligning programmes with well-known, preexisting Ministerial models of healthcare*, similar to other Ministerial programmes, to support programme sustainability.

**Table 4:** ‘Statements’ and examples of proposed outcomes

Statements (Policy recommendations)	Examples of proposed outcomes
1. What works in including persons with disabilities in decision-making regarding the development, implementation and monitoring/evaluation of policies/plans?	
1. Implementing the UNCRPD requires persons with disabilities to be involved in developing, implementing and evaluating rehabilitation policies, and for the capacity of persons with disabilities to be increased to strengthen their involvement.	1. Supports responsiveness to needs, and shared control over agenda setting.
2. Disability desks and focal persons should be established in all government ministries. Where persons with disabilities have appropriate levels of expertise and understanding given the context, they should be preferred candidates.	2. Strengthens focus on disability issues.
3. As an interim measure to promote inclusion, there should be a quota of policymakers who are persons with disabilities, which could be filled by persons with disabilities who have appropriate training and qualifications.	3. Prioritizes rehabilitation and supports participation of persons with disabilities in policy development.
4. New and advanced leadership pathways, such as volunteer opportunities, service on boards/committees, and leadership development workshops, should be created for disability advocates to represent persons with disabilities in service governance roles.	4. Equips service-users with skills to participate in advocacy and policy planning.
5. Research for rehabilitation services should be conducted with a participatory ethos. This requires that the research skills of persons with disabilities be developed, that the ability of researchers to meaningfully involve persons with disabilities is developed, and that adequate resources are provided by governments to increase such education/skill development.	5/6. Allows persons with disabilities to gain influence over research that guides policies.
6. More ‘emancipatory research’, or participatory research, should be conducted, allowing persons with disabilities to gain greater influence over decision-making for policies.	
7. Helping representatives of different types of disabilities to identity and express common challenges could strengthen their influence in service provision and ensure service provision responds to the full range of the diversity of disability.	7. Strengthens advocacy.
8. Service users of rehabilitation services should also be involved in the governance of such services, including for example on advisory and review panels and boards of steering committees.	8. – Strengthens programme sustainability. – Improves relevance of programmes.
9. ICT (information and communication technologies) are promising technologies for persons with disabilities to participate in e-governance in the long-term, including planning and monitoring.	9. Supports participation of persons with disabilities in governance.
10. Regular community analyses, context surveys, and user needs assessments are necessary to ensure that e-governance meets the needs of persons with disabilities.	10. – Assesses needs of subgroups of persons with disabilities to participate in e-governance. – Creates a comprehensive system design.
11. Statistical information and training should be available and accessible to persons with disabilities and DPOs so that they can meaningfully contribute to and engage with rehabilitation policy processes.	11. Creates a sense of ownership of research for persons with disabilities.
12. The participation of persons with disabilities, their families and their representatives in the planning, evaluation and monitoring of rehabilitation services should be mandated at local, national, regional and international levels.	12. – Supports service-user satisfaction. – Supports service efficiency/effectiveness.
2. What are the features of national legislation/policies that work to support the development and provision of rehabilitation services?	
13. A State’s Constitution and antidiscrimination laws should facilitate the realization of disability rights.	13. Strengthens legal and policy support for persons with disabilities and service-users.
14. It is critical that measures to support accountability and transparency in the provision of rehabilitation services are indicated in policies.	14. Supports accountability/transparency, so that governance creates inclusive, responsive and fair processes and outcomes, and public trust in a social system.
15. Rehabilitation should be integrated into general health policy and health sector reform plans, from primary care to tertiary hospitals with focus beginning on primary care.	15. Supports programme continuity.
16. CBR policies should be incorporated within existing health systems and with local and national health policies and legislation to ensure continuity and to secure annual budgets and other resources, while still allowing for a degree of flexibility of CBR projects.	16. Strengthens programme continuity and securing of resources for CBR.
17. Policies relating to rehabilitation should uphold the following seven primary aims for the provision of rehabilitation services (17–23 below): *Safe*: Avoid injury to people, including physical or psychological harm, from the care that is intended to help them.	17. Service-users avoid injury from care.
18. *Effective*: Provide services based on available scientific evidence to all who could benefit and refrain from providing services to those not likely to benefit.	18. Service-users receive appropriate care based on scientific evidence.
19. *Person centred*: Provide care that is respectful of and responsive to individual preferences, needs and values and ensure that service-users’ values guide all practitioners’ decisions. Awareness raising and education of service-users with regard to treatment options and human rights is important.	19. – Service-users receive appropriate, respectful and understanding care. – Service-users exercise choice.
20. *Timely*: Reduce waits and potentially harmful delays for both those who receive and practitioners who provide care.	20. Reduces waits for services.
21. *Efficient*: Avoid waste, including waste of equipment, supplies, ideas, and energy and take into account views and suggestions of service-users and their families.	21. Creates a structured system that matches resources with service demands.
22. *Equitable*: Provide care that does not vary in quality due to personal characteristics, such as gender, ethnicity, geographic location, socioeconomic status or type of impairment.	22. Supports justly distributed service provision based on need, including for vulnerable groups.
23. *Accessible*: Provide care that is accessible to all, including vulnerable groups, such as ethnic minorities, with regards to physical, economic, and information access to health services.	23. Strengthens accessible health care.
3. Do any of the listed features of national legislation and policies have a greater risk of adverse effect on particular groups of people and types of services?	
24. Policies should recognize that disability may interact with other vulnerability factors that increase discrimination, e.g. women or children with disabilities.	24. Supports access to services for persons with disabilities who may experience double discrimination and multiple disadvantages (e.g. ethnic minorities with disabilities).
25. Policies relating to rehabilitation should ensure that services are available to all groups of persons with disabilities, and allow disaggregation of data by subgroups that may be more vulnerable.	25. Supports access to services for all subgroups of persons with disabilities, such as persons with intellectual disabilities.
26. To promote equitable and accessible rehabilitation services, policies should specify how the particular barriers that marginalize certain groups would be overcome and associated budgetary allocation plans should be defined.	26. Supports access to services for vulnerable groups, such as children with special needs.
27. In national policies, specific mechanisms of exclusion in accessing health services should be addressed for different subgroups of persons with disabilities.	27. Policies support human rights and social inclusion in service provision.
28. The participation of persons with severe or multiple disabilities and persons with mental disabilities and/or their families/representatives in policy development should be prioritized/emphasized on an equal basis with others, with priority in contexts where they are significantly excluded from policy development.	28. Strengthens inclusion of subpopulations of persons with disabilities, such as persons with mental disabilities, who experience specific barriers to accessing services.
4. What are the features of a rehabilitation strategy/plan that work to achieve rehabilitation objectives?	
29. A national Rehabilitation Plan should be in place, and developed based on the UNCRPD, other international human rights instruments, and needs based assessments, with clear implementation and monitoring protocols.	29. Strengthens policy implementation.
30. CBR should be implemented by mobilizing partnerships, which include CBR programmes, government Ministries, persons with disabilities and their families and representatives, DPOs and NGOs.	30. Creates shared funding, resources, expertise, and ownership of programmes.
31. Strong inter-sectoral coordination, including coordination of funding, for all health related rehabilitation services, including CBR, is important with regards to provision of rehabilitation services.	31. Creates coherent mandates across governmental departments for services.
32. Health related rehabilitation should be integrated into a broader and comprehensive strategy to provide services for people who need rehabilitation services and persons with disabilities in all aspects of society, including health, employment, and education.	32. Strengthens access to services in all aspects of society for service-users.
5. What are the key steps to developing national legislation/policies and related strategies/plans for rehabilitation?	
33. Policymakers should receive rights based education/training to adopt a disability lens in the formation of all relevant policies.	33. Improves status and prioritization of rehabilitation amongst policymakers*.*
34. Governments should proactively consult with persons with disabilities, their families, DPOs, the private sector, NGOs, and international organizations throughout policy development.	34. – Supports service effectiveness. – Increases service-user satisfaction.
35. National authorities should align policy objectives and implementation with international instruments concerning the rights of persons with disabilities, such as the UNCRPD.	35. Provides a holistic approach for policies as the UNCRPD covers broad needs of service-users.
36. Mechanisms for sharing of information and experiences between countries and across regions should be strengthened for the purposes of national, regional, and local policy development.	36. Strengthens shared learning regarding service provision and policy development.
37. Information collected on disability should be disseminated proactively, succinctly, quickly, and in a language and format that decision-makers, as well as persons with disabilities, can easily and quickly understand.	37. Strengthens participation of persons with disabilities in decision-making.
6. What factors facilitate or impede the implementation of national legislation/policies and related strategies/plans for rehabilitation?	
38. A national Implementation Plan should be devised to support the implementation of policies for rehabilitation. Where a Rehabilitation Board exists, it should contribute to devising the plan.	38. Strengthens policy implementation.
39. A coordination mechanism, such as a National Disability Board, should be established to oversee the implementation of rights of persons with disabilities.	39. Oversees policy implementation, and coordinates national inter-sectoral liaison on disability.
40. A national Code of Practice should be formulated through input from service-users and aligned with the UNCRPD to implement policies on rehabilitation.	40. Supports implementation of policy and legislation; harmonizes public health laws.
41. Development of strategic alliances between the Rehabilitation unit and PCD of governments is important for the equitable implementation of policies for rehabilitation.	41. – Supports shared strengths/resources. – Creates co-ownership of a programme.
42. The alignment/integration of rehabilitation programmes with well recognized, preexisting models of healthcare delivery within the MoH can strengthen programme delivery and the implementation of policies for rehabilitation.	42. Supports programme sustainability.
43. Governments should provide equitable and nondiscriminatory levels of resources to implement policies for mental health services.	43. Promotes realizing rights in the lives of mental health service-users.
44. All government Ministries should have budget allocations to make services inclusive and accessible.	44. States comply with Article 9 of UNCRPD.
45. Governments should provide adequate funding and resources within their budgets to ensure the availability of human resources for implementation of policies for rehabilitation.	45. Provides sufficient numbers of trained rehabilitation workers.
46. CBR implementation is dependent on the support of community leaders, government, and persons with disabilities, DPOs, NGOs, rehabilitation professionals and paraprofessionals and the community.	46. – Increases CBR sustainability. – Enhances skills of those working in CBR.
7. What works in monitoring and evaluating rehabilitation legislation/policies and strategies/plans?	
47. National, regional and local Mental Health Review Boards should be in place to support mental health service-users and the provision of mental health services with participation of/contributions by service-users if prioritized by representative organizations in each context.	47. – Oversees policy implementation, and coordinates inter-sectoral liaison. – Protects the rights of rehabilitation service-users by investigating abuse and exploitation.
48. Governments should provide adequate levels of funding for the collection of disability statistics using both quantitative and qualitative research methods, including disaggregated information, to enable a situational analysis of disability.	48. *–* Informs planning. – Creates political momentum by identifying successful interventions.
49. A well-developed and well-implemented health management information system, which includes the collection of disability disaggregated data, should be in place with ethical privacy rules for management of data.	49. – Supports policymaker decision-making. – Assists evaluation of CBR programmes.
50. Government national, regional, and local CBR focal persons should be in place and regularly monitored.	50. Oversees CBR programmes.
51. A continuous review of processes is critical to identify areas of success and failure of any part of the process of the development, implementation and monitoring of policies.	51. Reviews policies to identify strengths and lapses in response to changes in demands, needs of service-users, and research findings.

## Discussion

A variety of broad principles emerged throughout formulation of recommendations: participation of persons with disabilities in policy processes; collection of disaggregated disability statistics; explicit promotion in policies of access to services for all subgroups of persons with disabilities and service-users; robust inter-sectoral coordination; and ‘institutionalizing’ programmes by aligning them with preexisting Ministerial models of healthcare.

An innovative methodology was used for this research that combined evidence from the literature with opinions of expert stakeholders to provide recommendations on policy for leadership and governance of health related rehabilitation in less resourced settings. These recommendations do not provide a prescriptive or exhaustive list, but propose broad principles that may be applied with consideration of contextual complexity and diversity. It is not appropriate, therefore, to propose how policy for governance of health related rehabilitation should be structured across all contexts. However, some general principles are provided, which may be valuable across diverse health systems and contexts, regarding optimal policy for governance of health related rehabilitation in less resourced settings.

Overall, findings from this research propose the meaningful participation of persons with disabilities in policy processes, and the research that guides such processes, as stipulated in the UNCRPD [16] and echoed pervasively in the literature [2, 3, 6, 14, 31, 32, 77, 79, 80]. It is also evident from this research that there may be concrete, measureable ways to strengthen participation, including supporting access to research and statistical information and training for persons with disabilities to support meaningful contribution of persons with disabilities to policy processes. Findings from this research suggest that outcomes of participation comprise strengthened self-determination; improved responsiveness to persons with disabilities’ needs and service-user satisfaction; enhanced efficiency, effectiveness, relevance, and sustainability of services; and shared ownership and influence regarding resources, processes, and outcomes of research that impact decision-making of policies.

The research findings also support the collection of disaggregated disability statistics, and development of health management information systems, to enable a situational analysis of disability, as per the UNCRPD [16], and literature [32, 76, 78]. As proposed by the World Report on Disability, ‘evidence for the effectiveness of interventions and programmes is extremely beneficial to guide policymakers in developing appropriate services; allow rehabilitation workers to employ appropriate interventions; (and) support people with disabilities in decision-making’ (pp. 121) [11]. According to our findings, robust data collection can inform decision-making of policymakers; instigate political momentum; strengthen evaluation and accountability of programmes; and enable equitable allocation of resources. Importantly, the research findings suggest that the value and impact of such data may be strengthened when disseminated proactively, succinctly, quickly, and in a language and format that decision-makers, as well as persons with disabilities, can easily understand. Furthermore, our findings indicate that mechanisms for sharing of data between countries and across regions may support shared learning regarding service provision and policy development.

Explicit recognition in policies that disability may interact with other vulnerability factors, for example displaced populations with disabilities, which may create double discrimination, multiple disadvantages, and increased barriers to accessing health services, is also supported by the research findings, and specified in the UNCRPD [16] and literature [53, 76, 80–82]. The findings also suggest that policies include disaggregated disability data and explicitly promote the availability of services for all subgroups of persons with disabilities, such as persons with intellectual disabilities. Thus, to promote inclusive, equitable and accessible rehabilitation services, it is recommended that policies specify how the particular barriers that marginalize specific groups will be overcome and define associated budgetary allocation plans. If social inclusion underpins policy formation, it is more likely that this principle will be inculcated in health service delivery [6, 79].

Strong inter-sectoral coordination for the provision of rehabilitation services, including CBR, is supported by the research findings, for the purposes of creating coherent mandates across governmental departments regarding service provision, as reflected in the UNCRPD [16], World Report on Disability [11], Declaration of Alma-Ata [14], and literature [42, 53, 76, 77]. Coordination is essential as disability is a crosscutting issue [67, 70]. In accordance with our findings, a coordination mechanism is proposed, such as a National Disability Board or Mental Health Review Board, to coordinate inter-sectoral liaison on disability and rehabilitation at national, regional and local levels. Through establishing a coordinating mechanism, States may comply with Article 33 on ‘National implementation and monitoring’ of the UNCRPD [16], and ensure that responsibility for realizing the rights of persons with disabilities encompasses an extensive range of governmental sectors at different levels. Our findings also support the integration of health related rehabilitation into a broader and comprehensive strategy to strengthen inclusion and access to services for service-users and persons with disabilities in all aspects of society, including health, employment, and education, as advocated by the CBR Guidelines [15] and UNCRPD [16].

Programme ‘institutionalization’ can be realized by aligning a rehabilitation programme with well-known, preexisting models of healthcare, akin to those of other Ministerial programmes, to support programme sustainability, as indicated by this research [42, 76]. Comparable mechanisms to support programme sustainability, also suggested by our findings, include the integration of rehabilitation programmes into a national system, such as the MoH and other relevant Ministries, so that a programme continues to function, irrespective of the cessation of donor funding for example. Similarly, ‘institutionalizing’ policies for CBR with local and national policies and legislation can support continuity.

Aside from the formal health system, governance comprises collaboration with other sectors, including the private sector and civil society, to promote population health in a way that is participatory and inclusive [83]. As emphasized by Siddiqi et al. [2], good governance does not concern governments alone, but comprises the ‘complex mechanisms, processes and institutions through which citizens and groups articulate their interests, mediate their differences and exercise their legal rights and obligations’ (pp. 14). Accordingly, in addition to national policymakers, our policy recommendations have relevance for a variety of stakeholders, including international nongovernmental organizations, intergovernmental organizations, private sector, DPOs, civil society, national service providers, community service providers, service-users and their representatives and families; our recommendations are supported by evidence collected on, by and for such stakeholders. Our recommendations recognize the importance of extensive, careful and meaningful collaboration between such stakeholders throughout policy processes, and the research that guides such processes. Moreover, translating research into evidence-based policy needs to be systematically addressed using a coordinated and coherent approach with the inclusion of a wide range of stakeholders [71]. Just as individuals perceive environmental threats and opportunities differently [84], so too stakeholders perceive different threats and opportunities in the policy environment and these need to be addressed in order to keep stakeholders engaged, empowered and supportive.

### Strengths and limitations

By using a realist synthesis to explore policy for leadership and governance of health related rehabilitation in less resourced settings, evidence and insight on this research topic has been generated that would most likely not be provided by alternative empirical approaches. Combining a realist synthesis with a Delphi study offered a unique approach to synthesis. This two-method study design offered the advantages of combining the authority and contextual focus of a systematic search and realist synthesis of the literature, with the additional credibility of increased stakeholder expertise and experience provided by the Delphi survey.

One challenge, however, was the broad research topic, which necessitated narrowing our research question and literature search to conduct more focused and informative research. Furthermore, combining the opportunities of a Cochrane style systematic search of the literature with a realist synthesis was at times challenging. For example, a common characteristic of systematic reviews, including Cochrane reviews [85], is formal appraisal of the methodological quality of included studies [86]. However, as outlined above, as realist syntheses include a wide variety of documents, the methodological quality rating of several articles could not be appraised.

The Delphi method also has a number of observed limitations. For example, the panel of experts, while purposefully chosen to fulfill selected criteria of diverse stakeholder perspectives, was nonetheless dependent on our own networks and this may have introduced sampling bias. Furthermore, interactions between the researcher and participants were not in person but computer mediated and this may have influenced the gathering of information. The participation of experts from different countries and health systems with diverse priorities and resources may have increased the difficulty of arriving at consensus [40]. Importantly, however, diversity of opinions may reflect the variety of approaches in international health related rehabilitation practice [40] and policy processes. Indeed, this Delphi study availed of a diverse range of experts to confirm important elements of health related rehabilitation policy processes in varied contexts, and so we effectively built in heterogeneity to the study participant pool.

This research highlighted that little direct evidence is available on successful policy for leadership and governance of health related rehabilitation in less resourced settings. This dearth of evidence suggests that researchers and decision-makers collect and disseminate more robust and detailed evidence to support this area of research and practice.

To the best of our knowledge, there are no previously conducted realist studies on policy for governance of health related rehabilitation in less resourced contexts. However, the findings are in accordance with other health policy and strategy focused studies in their support of contextualizing health policies and policy processes [20, 22].

## Conclusions

This research aims to enable 'guided transformation' of policy for leadership and governance of health related rehabilitation by proposing broad recommendations, which require contextual adaptation. Importantly, there is no one-size-fits-all approach to reforming health care systems; rather, policymakers can use principles of best practice to increase the effectiveness of health care spending and the efficiency of health care systems [87]. The innate complexity and substantial diversity across health systems and broader socio-geo-political contexts necessitates more general and sufficiently flexible policy recommendations. Accordingly, our study proposes broad principles of successful policy for leadership and governance of health related rehabilitation in less resourced settings.

## Abbreviations

AIM, african index medicus; C, context; CAHD, community approaches to handicap in development; CAS, complex adaptive systems; CBR, community based rehabilitation; CMOCs, context mechanism outcome pattern configurations; DPOs, organizations of persons with disabilities; ICT, information and communication technologies; ILD, inclusive local development; LMIC, low and middle-income countries; M, mechanism; MMAT, mixed methods appraisal tool; MoH, ministry of health; N/A, not applicable; NGO, non-governmental organization; O, outcome; PCD, primary care division; PNDTD, (programa nacional de diagnóstico y tratamiento de la depresión) national depression detection and treatment program; UNCRPD, united nations convention on the rights of persons with disabilities; WHO, world health organization
